# How Negative Is Negative Information

**DOI:** 10.3389/fnins.2021.742576

**Published:** 2021-09-07

**Authors:** Elisabeth Simoes, Alexander N. Sokolov, Markus Hahn, Andreas J. Fallgatter, Sara Y. Brucker, Diethelm Wallwiener, Marina A. Pavlova

**Affiliations:** ^1^Department of Women’s Health, University Hospital, Eberhard Karls University of Tübingen, Tübingen, Germany; ^2^Executive Department for Social Medicine, University Hospital, Eberhard Karls University of Tübingen, Tübingen, Germany; ^3^Department of Psychiatry and Psychotherapy, Medical School and University Hospital, Eberhard Karls University of Tübingen and Tübingen Center for Mental Health (TüCMH), Tübingen, Germany

**Keywords:** negative information, neural networks, devastating events, social cognition, social reasoning, depression and anxiety, breast cancer, gender impact

## Abstract

Daily, we face a plenty of negative information that can profoundly affect our perception and behavior. During devastating events such as the current COVID-19 pandemic, negative messages may hinder reasoning at individual level and social decisions in the society at large. These effects vary across genders in neurotypical populations (being more evident in women) and may be even more pronounced in individuals with neuropsychiatric disorders such as depression. Here, we examine how negative information impacts reasoning on a social perception task in females with breast cancer, a life-threatening disease. Two groups of patients and two groups of matched controls (*N*_TO__TAL_ = 80; median age, 50 years) accomplished a psychometrically standardized social cognition and reasoning task receiving either the standard instruction solely or additional negative information. Performance substantially dropped in patients and matched controls who received negative information compared to those who did not. Moreover, patients with negative information scored much lower not only compared with controls but also with patients without negative information. We suggest the effects of negative information are mediated by the distributed brain networks involved in affective processing and emotional memory. The findings offer novel insights on the impact of negative information on social perception and decision making during life-threatening events, fostering better understanding of its neurobiological underpinnings.

## Introduction

Every day we face a plenty of information with emotional relevance that we perceive as positive or negative. Messages along this positive—negative dimension bring about reward or threat with profound effects on perception and behavior (Spencer et al., 2016; Gu et al., 2019; Martins et al., 2021). Consider, for example, common reminders of cognitive and, in particular, memory decline with aging that yield a greater drop of performance in elderly compared to when the reminder is absent (e.g., Barber, 2017). By contrast, providing peer encouragement as a positive feedback to children prior to a challenging physical task is associated with more positive reports of self-efficacy and improved performance (e.g., Innes et al., 2020).

During devastating events such as the current coronavirus (COVID-19) pandemic, negative messages are often unavoidable and may hinder problem solving at individual level and social decisions in the society at large. Moreover, positive messages during the pandemic are reported to be associated with better resilience ameliorating the negative impact of the present crisis, especially for those experiencing negative emotions (Israelashvili, 2021). Influence of negative information may be even more pronounced in individuals with neuropsychiatric disorders, most of which are gender specific (Pavlova, 2017). Recent evidence indicates gender (as a social construct)/sex (as a neurobiological construct) specificity in the way and degree of the pandemic impact on depression: it affects females more heavily than males. For instance, recent data on the mental health burden in the German population during the COVID-19 pandemic indicates significantly increased symptoms of generalized anxiety (44.9%), depression (14.3%), psychological distress (65.2%), and fear (59%), with females and younger people reporting higher mental burden (Bäuerle et al., 2020). Women more often than men use hotlines on virus-related health concerns and corresponding facilities (e.g., Peters et al., 2020; Wenham, 2020). In Chinese individuals examined during the COVID-19 pandemic, women encounter more severe stress and anxiety symptoms than men (Hou et al., 2020). Similarly, in the Turkish population (apart from a history of mental illness), *being female* has become a risk factor for anxiety and depression (Özdin and Bayrak Özdin, 2020).

Negative messages not only affect perception, emotion, and behavior, but also modulate brain activity (Wraga et al., 2007; Krendl et al., 2008; Cikara et al., 2011; Forbes and Leitner, 2014; Forbes et al., 2018). For example, performing demanding cognitive tasks (such as mental rotation and math problem solving) under influence of negative information leads to functional magnetic resonance imaging (fMRI) activation of the brain regions underwriting executive control and affective processing (e.g., the amygdala and ventral anterior cingulate cortex, ACC; Wraga et al., 2007; Krendl et al., 2008) that block cognitive processes and lead to a decrease in performance. By contrast, with positive information, the same tasks engage task-related brain regions (such as the medial temporal gyrus, MTG, and ventral anterior prefrontal cortex, PFC) underpinning visual spatial skills and working memory (Wraga et al., 2007). In avid fans of renowned baseball teams, positive outcomes such as success of the favored team activate in fMRI the ventral striatum associated with subjective pleasure, whereas negative outcomes (e.g., failure of the favored team) engage the ACC and insula (Cikara et al., 2011). A negative feedback on a task increases amygdala activation and emotional memory network connectivity (Forbes et al., 2018).

The ventromedial PFC (vmPFC) is implicated in processing of negative messages as patients with lesions to these regions are less affected by negative information than healthy controls (Milne and Grafman, 2001). The underwriting networks seem to be sex-specific: bilateral prefrontal transcranial magnetic stimulation (TMS) increases the effect of gender-related negative messages in males, but not females (Cattaneo et al., 2011). Overall, positive messages affect brain function by recruiting efficient neural networks that support cognitive tasks at hand, whereas negative information engages areas involved in negative-emotion processing that inhibit task-specific networks. Negative information also modulates sex hormone levels, for example, testosterone levels of males in gender-stereotype-activated group performing cognitive tasks are 60% higher than those of controls (Hausmann et al., 2009). This is of importance for health-related issues and especially, diseases that depend on hormone circulation.

Generally, positive messages given prior to task administration enhance subsequent performance whereas negative messages worsen performance (Pavlova et al., 2014; Forbes et al., 2018). These effects vary substantially across genders: They are shown to be more pronounced in females with a greater impact of negative messages even on the tasks with no genuine gender differences when receiving a standard neutral instruction (Pavlova et al., 2010, 2014). Recent work suggests that similar unfavorable effects occur in patients (*diagnosis threat*; Suhr and Gunstad, 2002, 2005; Kit et al., 2008, 2014; Blaine et al., 2013; Pavawalla et al., 2013). During counseling, disease-related negative information can deteriorate not only patients’ performance on neuropsychological and cognitive tests, but also interfere with their reasoning as to potentially best strategies of drug and surgery treatment.

The present work is directed at examination of possible effects of negative information in gynecological oncologic diseases such as breast cancer, the second most common type of cancer among women (Robert Koch-Institut, 2017; American Cancer Society, 2019). Breast cancer is well-known to represent a significant threat to affected individuals (e.g., Keyzer-Dekker et al., 2014). When diagnosed with breast cancer, women face a lot of frightening information that may substantially hinder their perceptual and cognitive abilities, eventually resulting in gender-specific (and often suboptimal) coping with the disease. We intend to assess whether, and if so, how, negative messages impact performance on social perceptual tasks in female breast cancer patients.

## Materials and Methods

### Participants

Eighty women (40 female patients with primary breast cancer recruited on a voluntary basis from the Department of Women’s Health, University Hospital of Tübingen, and 40 matched control females from the local community) participated in the study. The inclusion criteria for patients were a first diagnosis of breast cancer (diagnosis code according to ICD-10-GM, C50—Malignant neoplasm of breast), no prior or current chemotherapy, metastases, no any other oncologic, neurological or psychiatric diseases as documented in the patient records (Table 1). The age of breast cancer patients and control participants was in the range of 39–58 years with a median of 50 years. In order to attain the most homogeneous study population, women who prior to the initial diagnosis, had attended breast cancer screening were excluded. This reduced potential biases related to variable anamnesis (such as screening-based initial diagnosis and tedious patient careers). Participants were assigned to one of four groups of 20 persons each (see below and Table 1). Participants of the control groups were matched to the patients regarding their sociodemographic parameters including age and educational status.

**TABLE 1:** Summary of participant characteristics.

Group	Age in years (mean ± SD)	Inclusion criteria	Exclusion criteria	Recruitment
**Female breast cancer patients** **Two groups** à 20 persons • *With* negative information (PAT*NI) • *Without* negative information (PAT)	48.95 ± 5.20 49.90 ± 4.47	First diagnosis (diagnosis code according to ICD-10-GM, C50 – Malignant neoplasm of breast)	Any neurological and/or psychiatric comorbidity Prior and/or current chemotherapy Secondary metastases and/or breast cancer in progress Failure to secure consent	Female patients of the Breast Centre, University Women‘s Hospital, Department of Women’s Health, University Hospital Tübingen
**Female control participants** **Two groups** à 20 persons • *With* negative information (CTR*NI) • *Without* negative information (CTR)	47.89 ± 6.51 50.05 ± 5.07	Person-by-person matched to the patients with respect to age and sociodemographic parameters	Any neurological and/or psychiatric disorders Prior and/or current oncologic disease Prior and/or current chemotherapy Failure to secure consent	Local population

Two groups of participants [patients, PAT: age, 49.90 ± 4.47 years (mean ± SD, standard deviation); and healthy control females without oncologic diseases in their history, CTR: age, 50.05 ± 5.07 years] were given only a standard instruction prior to the test. The experimental patient group (PAT^∗^NI: age, 48.95 ± 5.20 years) received, along with the standard instruction, gender-related negative information (see below). Another group of control females (CTR^∗^NI: age, 47.89 ± 6.51 years) also received, similar to the experimental patient group, prior negative information. Including the patient and control groups with and without negative information allowed us to disentangle factors Disease and Negative Information. All four groups were comparable in respect to the age of participants [a one-way analysis of variance, ANOVA, *F*_(3,78)_ = 0.675, *p* = 0.570, *n.s.*].

All participants had normal or corrected-to-normal vision and were run individually. None had previous experience with such tasks. The study was conducted in line with the Declaration of Helsinki and approved by the local Ethics Committee at the University of Tübingen Medical School. Informed written consent was obtained from all participants. Participation was voluntary, and the data were processed anonymously. Upon completion of the experiment, participants received a small chocolate box that was unexpected before examination.

### Task and Procedure

The Event Arrangement (EA) test was administered to participants. The task is included in the *Wechsler Intelligenztest für Erwachsene* (WIE), a test battery based on the WAIS-III (Wechsler Adult Intelligence Scale-III) by David Wechsler adapted to the German population (von Aster et al., 2006). The task was described in more detail elsewhere (Pavlova et al., 2010). In brief, the EA task is a well-established tool for psychological assessment, psychometrically standardized, and provides normative scores obtained from a large population. For this task, participants are administered 11 sets of cards portraying human characters, their actions, and interactions. The sets differ in the number of cards and their complexity. Each set is presented in a predetermined scrambled (false) order. The participants have to rearrange cards into a correct order depicting an event in a comic-strip fashion, thereby showing understanding of the event. It is assumed that performance on such tasks requires understanding the characters’ mental states and interactions, and, therefore, taps social cognitive capabilities (Baron-Cohen et al., 1986; Völlm et al., 2006; Pavlova et al., 2008). For successful performance, participants need to reflect the core of the story, which is often based on veridical perception of intentions and drives of the characters involved in this particular event. Both accuracy (correct order of cards in a sequence) and the time needed for an event arrangement (as a specific time frame for each set defined *a priori* according to the event complexity) are taken into account when assessing performance on the task. Participants are told that the sets have specific time frames for their rearrangement.

For each set, a raw value (2 through 0) is assigned according to either correct, acceptable or incorrect card order attained by the participant as given in the WIE Manual (von Aster et al., 2006). A total raw value per participant is computed as a sum across all sets. With the help of the WIE Manual tables that take into account participants’ age, the raw values are then transformed into the standardized normative scores ranging from 1, floor performance, through 10, normal or typical performance for this age, to 19, ceiling performance.

Prior to completing the test, participants of all groups received a standard instruction on how to perform the task. In addition to the standard instruction, the experimental patient group (PAT^∗^NI) and one control group of healthy participants (CTR^∗^NI) were told that men usually perform better than women on this task (an implicit negative message for females, whereas an explicit negative message would be *women usually perform worse than men on this task*). The reasons for choosing this kind of negative information in the present study were threefold: (i) such messages had already been used previously in healthy (albeit younger) females (Pavlova et al., 2010, 2014), and shown to substantially affect their performance on the same task, (ii) delivery of any kind of disease-related negative information to patients in an experimental laboratory setting would be ethically doubtful, and (iii) disease-related messages would be hardly applicable with healthy control participants. Moreover, it has been suggested that gender-related threat and diagnosis threat are similar as to their effects and ways of action (Schmader et al., 2008).

Prior to the task, all groups also filled in short surveys on gender identification, which was primarily intended to activate the participants’ gender identity. Gender identification items were borrowed from a standardized tool for socioeconomic panel surveys targeting German population and routinely run by the German Institute for Economic Research (Siedler et al., 2009; DIW/SOEP, 2011). Specifically, the survey administered in this study included a general 11-point scale asking each participant to rate her overall self-estimation as a woman as compared to other women (−5, *much less feminine*, through 5, *much more feminine*), and a number of subscales for self-estimation of participants’ femininity in daily life situations: while being alone, among friends, relatives, and strangers (for example, “How feminine do you feel compared to other women, when you are among family members?”). Patients were also asked to answer several questions on the potential impact of disease taken from a standardized questionnaire EORTC QLQ-C30 for assessment of quality of life in cancer patients (Version 3 adapted for German population; EORTC Quality of Life Group, 2011). The whole procedure had to be adapted to the restricted capabilities of the patients (Wallwiener et al., 2016) and, therefore, a session was completed within about half an hour.

Prior to all statistical analyses, normality of data distributions was routinely assessed by Shapiro-Wilk tests with subsequent uses of either parametric (for normally distributed data sets) or, otherwise, non-parametric statistics.

## Results

All four groups of participants did not differ on their median gender identification scores (Kruskal-Wallis test, *H_3_* = 6.25, *p* = 0.10, *n.s.*). Most important, self-estimation of femininity in all four groups of participants was not only statistically the same, but in all groups, females rated themselves as feminine as other women. No difference was found between two patient groups on their median scores for self-reported burden of disease (two-tailed Mann-Whitney test, *U* = 249, *p* = 0.19, *n.s.*).

**FIGURE 1:**
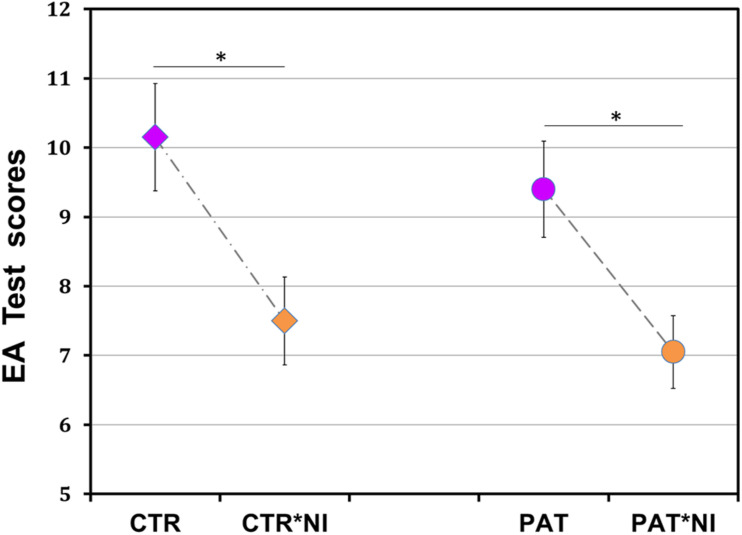
Unfavorable effect of negative information on female breast cancer patients’ social reasoning ability. Performance on a social reasoning (event arrangement, EA) task was substantially reduced in both patients and matched control participants who received prior negative information compared to those who did not (*p* < 0.0001). Significant effects of information in groups of patients (*p* < 0.03) and controls (*p* < 0.01) are shown by asterisks. PAT*NI, PAT: patients either with or without negative information; CTR*NI, CTR: matched control females either with or without such information. Total score range, 1–19; normal score, 10. Each data point, mean ± SEM, standard error of mean.

Figure 1 shows the mean EA test scores for four groups of participants along with respective variability measures. Individual test scores were submitted to a two-way ANOVA with the between-subject factors Negative Information (yes/no) and Disease (yes/no). The outcome yielded a highly significant main effect of Negative Information [*F*_(1,76)_ = 18.29, *p* < 0.0001; effect size, *eta squared* η^2^ = 0.29]. Performance was generally worse in the groups of patients and matched controls who received negative information compared to those who did not receive such information. Neither main effect of Disease [*F*_(1,76)_ = 1.05, *p* = 0.308, *n.s.*] nor Disease by Negative Information interaction were found [*F*_(1,76)_ = 0.07, *p* = 0.798, *n.s.*].

As expected from earlier work (Pavlova et al., 2010, 2014), *post hoc* analyses revealed a significant difference between control participants with and without negative information (mean ± SD, 7.50 ± 2.37, 95% confidence interval, CI [6.46; 8.54] versus 10.15 ± 2.89, 95% CI [8.88; 11.42], with lower scores for individuals with negative information: two-tailed Tukey’s honestly significant difference, HSD test corrected for multiplicity throughout, *t*(38) = 3.21, *p* < 0.01; effect size, Cohen’s *d* = 1.00).

Most important, under the influence of negative information, patients scored not only lower than control participants with neutral standard instruction (7.05 ± 2.28, 95% CI [6.05; 8.05], compared to 10.15 ± 2.89, 95% CI [8.88; 11.42]; *t*(38) = 3.75, *p* < 0.002; effect size, *d* = 1.19), but also lower than patients without such negative information (7.05 ± 2.28, 95% CI [6.05; 8.05], compared to 9.40 ± 2.85, 95% CI [8.15; 10.65]; *t*(38) = 2.84, *p* < 0.029; effect size, *d* = 0.91). No differences occurred between the groups of patients and controls without negative information (9.40 ± 2.85, 95% CI [8.15; 10.65], compared to 10.15 ± 2.89, 95% CI [8.88; 11.42]; *t*(38) = 0.91, *p* = 0.801, *n.s.*), as well as between the patient and control groups with information (7.05 ± 2.28, 95% CI [6.05; 8.05], compared to 7.50 ± 2.37, 95% CI [6.46; 8.54]; *t*(38) = 0.54, *p* = 0.948, *n.s.*).

## Discussion

The present study examines whether and, if so, to what extent negative information affects social cognition and reasoning in a sample of females with a life-threatening disease such as primary breast cancer. Previous research reported unfavorable effects of negative information on social cognition and behavior in young healthy women, university students (Pavlova et al., 2010, 2014). Here, comparable adverse effects are documented in older healthy women, suggesting largely age-independent influence of negative information on social cognition and reasoning in females.

Most important, the outcome points to the unfavorable impact of negative messages on visual social cognition in females with breast cancer. In oncologic populations, negative health-related messages may significantly worsen patients’ objective situation and yield auxiliary mental health conditions (Keyzer-Dekker et al., 2013, 2014). Indeed, when diagnosed with such a devastating disease as breast cancer, women face a plenty of negative health- and gender-related information that may substantially hamper their cognitive abilities and decision making (e.g., during informed consent, treatment options assessment), eventually resulting in less optimal coping with disease.

The present work indicates that even in a snapshot laboratory setting, delivery of negative information yields a drastic decline in performance on a social cognition task in female breast cancer patients. Patients’ performance is substantially reduced as compared not only with performance of matched control individuals, but also with patients without negative information. The findings suggest that social cognitive reasoning in females with breast cancer is particularly susceptible to the effect of negative messages. This may considerably hinder the patients’ coping with the disease and ultimately, the best possible treatment outcome, even with a favorable course of the disease and encouraging prognosis of surgery and drug therapy.

The drop in performance associated with negative information was similar in patient and control groups: There was no interaction between the factors disease and negative information. This outcome may be accounted for by the time point of patient recruitment in the present study (shortly after surgery), when prevalence and levels of both anxiety and depression are reportedly decreased (e.g., Garssen et al., 2013; Kim et al., 2020). One may assume that at that period, a negative impact of the threatening diagnosis of breast cancer *by itself* on social cognition, reasoning, and behavior might be diminished, yielding a lack of interaction between the factors disease and negative information. In this light, the substantial effect of negative information on patients appears even more remarkable. It is known that compared to patients with benign breast disease (BBD) and gallstone disease, the breast cancer diagnosis *as such* can induce strong fatigue in affected individuals, largely diminishing their reported quality of life (Keyzer-Dekker et al., 2013). Moreover, this diagnosis yields an increase in state anxiety and depressive symptoms (Keyzer-Dekker et al., 2014; Aquil et al., 2021; Li et al., 2021; Thakur et al., 2021). Depression is a comorbid disorder to breast cancer, and if neglected, it may complicate the treatment of both illnesses, which can result in poor adherence to treatment and less desirable outcome (Thakur et al., 2021). This is of particular importance in light of the current COVID-19 pandemic leading to delayed surgery, disruptions in patient care, and threat of COVID-19 diagnoses (Soriano et al., 2021). Future research will examine the effects of information delivery at other critical time points in the course of disease, for instance, shortly upon the initial diagnosis or returning to daily life after surgery and completion of treatment (Simoes et al., 2016).

In developed countries, breast cancer is the second most common type of cancer in women, with over 260,000 new cases in the U.S. and 70,000 per year in Germany (Robert Koch-Institut, 2017; American Cancer Society, 2019). As due to recent advances in biomedicine, the survival rates for breast cancer rise, the issues of proper doctor-patient interaction, communication, and information delivery gain much greater attention to further improve treatment outcomes and patients’ quality of life (Jensen et al., 2014; Li et al., 2021). Patients’ choice of optimal therapy options and short-term and long-term outcomes (e.g., duration of hospital stays and need for analgesic drugs) can heavily depend on the way information is delivered to the patients (Arraras et al., 2007; Angioli et al., 2014; Thakur et al., 2021).

Diagnosis- and therapy-related information can modulate both the course of oncologic diseases and side effects of their therapy. When cancer patients undergoing chemotherapy are told about possible chemotherapy associations with certain cognitive dysfunctions (so called *chemobrain*; Gehring et al., 2012), they present greater cognitive complaints and also score worse on subsequent neuropsychological cognitive tests (Schagen et al., 2012). Similarly, once informed about forthcoming adjuvant hormone therapy, 25% of breast cancer patients develop negative expectations in this regard, yielding psychogenic side effects and poor therapy adherence (Heisig et al., 2016). At the same time, information on ways of action and potential side effects of a therapy can improve patient autonomy and therapy adherence, especially in older patients (Heisig et al., 2015). Up to now, however, the precise mechanisms of these effects remain poorly understood (Hutchinson et al., 2012).

So far, in male patients (e.g., with prostate cancer; Murray et al., 2015), only indirect indications of unfavorable impact of negative information exist. Healthy males are more susceptible to *explicit* rather than implicit negative information (Pavlova et al., 2014). As in the course of disease, much information during patient-doctor counseling has to be conveyed in explicit ways, this could likely affect male patients, substantially worsening their objective situation and hindering best possible treatment outcomes.

It is essential that not all individuals are equally affected by negative messages. Clarification of person-related factors (i.e., individual differences) modulating the detrimental impact of negative information represents a major challenge for future research. This is of particular value while striving for personalized medicine. In most critical events such as serious illnesses, epidemics and natural catastrophes (earthquakes, floods), delivery of negative information is common and unavoidable. This can adversely affect mental health in both single persons and entire populations, increasing the humanitarian burden and economic cost of the events to the society. An evidence-based, trustable, balanced and tailored communication of negative information is, therefore, vital for successful management of emergency cases and safeguarding the population’s mental health and economic strength in challenging times.

Brain mechanisms underwriting adverse effects of negative information are largely unknown. Wraga et al. (2007) and Krendl et al. (2008) report fMRI activation following negative message delivery in brain regions engaged in affective processing and executive control (e.g., the amygdala, ventral anterior PFC, and ACC). By contrast, without such messages (or with positive ones), brain activations comprise the networks of regions supporting particular functions tapped by the tasks at hand (e.g., visual perceptual networks). In passionate fans of celebrated U.S. baseball teams, subjectively negative outcomes (failure of the favored team or success of the rival team) activate in fMRI the ACC and insula, whereas positive outcomes (success of the favored team or failure of the rival team, even against a third team) activate the ventral striatum implicated in subjective pleasure (Cikara et al., 2011). During math problem solving, negative messages yield a greater midline P100 component of event-related potentials, ERPs, tighter phase locking between the ACC and dorsolateral PFC, dlPFC (two attention-related regions), and higher power in the left fusiform gyrus, FFG (Forbes and Leitner, 2014). In negative contexts only, the left FFG power is inversely predictive of participants’ task performance. In women (but not men), encoding of negative information is associated with amygdala activation and emotional memory network connectivity (Forbes et al., 2018). Non-verbal negative contexts induced during math problem solving increase automatic error vigilance (as indexed by error-related negativity, ERN) in female students from the science, technology, engineering, and mathematics, STEM, field: Those highly investing in their STEM careers display greater ERN upon making errors when primed by negative context images, whereas no such priming occurs in men (Wu et al., 2020).

**FIGURE 2:**
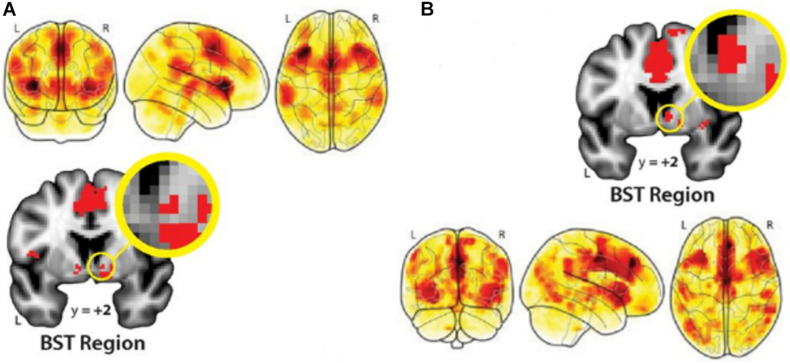
Distributed brain networks that underwrite processing threatening information in the human brain. **(A)**, Temporally certain and **(B)**, uncertain threat (versus respective safe conditions) activate essentially overlapping large-scale networks of such key brain regions as the amygdala, ACC, bilateral IFG, anterior insula, right superior temporal gyrus, and BST region. From Hur et al. (2020). Copyright © 2020 by The Society for Neuroscience; Creative Commons Attribution 4.0 International License (CC-BY).

Negative information appears to elicit a physiological stress response, with pathways connecting the ACC with both the amygdala and hypothalamus that in turn, trigger release of particular hormones and bodily reactions (LeDoux, 2000; Muscatell and Eisenberger, 2012). Most recent experimental work and meta-analyses of neuroimaging evidence reveal a large-scale neural functional architecture for threat processing in the human brain. Temporally certain threat (such as awaiting an oncologic diagnosis in a few days) and uncertain threat (fear of cancer being an incurable disease, changes in body image, and fear of death) engage essentially similar brain networks (Figure 2). These connectomes comprise such key structures as the midACC, bilateral inferior frontal gyrus/anterior insula/Rolandic operculum, dlPFC, periaqueductal gray (PAG), bed nucleus of the stria terminalis (BST), and amygdala (Hur et al., 2020; Chavanne and Robinson, 2021). Other brain regions engaged are the bilateral supramarginal, right superior temporal, right middle frontal, and right precentral gyri (Chavanne and Robinson, 2021). Future research will uncover the precise origins of the influence of negative information on brain function and behavior, involving distributed brain networks underwriting affective processing and emotional memory.

During devastating events such as the COVID-19 pandemic, communication of negative information is unavoidable. This can increase anxiety and exert unfavorable influence in gender-dependent ways, hindering problem solving at individual level and social decisions in the society. It is, therefore, worth taking a closer look at gender specificity (and generally, individual differences) of negative-information effects both in life-threatening diseases and global emergency conditions to generate properly tailored real-world applications.

Information delivery and communication are manageable. Negative effects of information and communication can adversely affect therapy and its outcomes in hazardous diseases and social decisions in emergency conditions. Once the underlying (including neurobiological) mechanisms of detrimental effects on the brain and behavior are known, one can target their causes in much more appropriate ways.

## Résumé

The present study examined whether negative information affects social cognition and reasoning in a hazardous disease such as female breast cancer. With negative information, patients scored not only lower than control participants, but also patients without negative information. For the first time, the results show a strong impact of negative information on visual social cognition and problem solving in patients with life-threatening diseases. Future research will uncover brain mechanisms of these effects. Other challenges include investigation of impact of negative messages in male patients and identification of person-related factors governing vulnerability to negative information across individuals. The findings offer novel valuable insights on perception and decision making under delivery of negative information during life-threatening events to foster better understanding of its detrimental impact, in particular, at the level of underlying brain mechanisms. This would make possible properly tailored real-world applications.

## Data Availability Statement

The raw data supporting the conclusions of this article will be made available by the authors to any qualified researcher, without undue reservation.

## Ethics Statement

The studies involving human participants were reviewed and approved by the Ethics Committee at the University of Tübingen Medical School, Tübingen, Germany. The patients/participants provided their written informed consent to participate in this study.

## Author Contributions

ES, MP, and AS designed the research conception, conceived and designed the experiments, and wrote the manuscript. AS performed the experiments. AS and MP analyzed the data and secured patients recruitment. MH, SB, and DW contributed to patient management. AS, AF, SB, DW, MP, and ES contributed to reagents, materials, and analysis tools. All authors contributed to the writing and editing of the manuscript.

## Conflict of Interest

The authors declare that the research was conducted in the absence of any commercial or financial relationships that could be construed as a potential conflict of interest.

## Publisher’s Note

All claims expressed in this article are solely those of the authors and do not necessarily represent those of their affiliated organizations, or those of the publisher, the editors and the reviewers. Any product that may be evaluated in this article, or claim that may be made by its manufacturer, is not guaranteed or endorsed by the publisher.
